# The Association between Chronic Widespread Musculoskeletal Pain, Depression and Fatigue Is Genetically Mediated

**DOI:** 10.1371/journal.pone.0140289

**Published:** 2015-11-24

**Authors:** Andrea Burri, Soshiro Ogata, Gregory Livshits, Frances Williams

**Affiliations:** 1 Department of Twin Research and Genetic Epidemiology, King’s College London, St. Thomas´ Hospital, London, United Kingdom; 2 Department of Psychology, University of Zurich, Binzmühlestrasse 14, 8050, Zurich, Switzerland; 3 Department of Health Promotion Science, Osaka University Graduate School of Medicine, Suita, 565–087, Osaka, Japan; 4 Sackler Faculty of Medicine, Tel Aviv University, Tel Aviv, 69978, Israel; NIH - National Institute of Environmental Health Sciences, UNITED STATES

## Abstract

**Background:**

Chronic widespread muscoloskeletal pain (CWP) is prevalent in the general population and associated with high health care costs, so understanding the risk factors for chronic pain is important for both those affected and for society. In the present study we investigated the underlying etiological structure of CWP to understand better the association between the major clinical features of fatigue, depression and dihydroepiandrosterone sulphate (DHEAS) using a multivariate twin design.

**Methodology/Principle Findings:**

Data were available in 463 UK female twin pairs including CWP status and information on depression, chronic fatigue and serum DHEAS levels. High to moderate heritabilities for all phenotypes were obtained (42.58% to 74.24%). The highest phenotypic correlation was observed between fatigue and CWP (r = 0.45), and the highest genetic correlation between CWP and fatigue (r_g_ = 0.78). Structural equation modeling revealed the AE Cholesky model to provide the best model of the observed data. In this model, two additive genetic factors could be detected loading heavily on CWP—A2 explaining 40% of the variance and A3 20%. The factor loading heaviest on DHEAS showed only a small loading on the other phenotypes and none on fatigue at all. Furthermore, one distinct non-shared environmental factor loading specifically on CWP—but not on any of the other phenotypes—could be detected suggesting that the association between CWP and the other phenotypes is due only to genetic factors.

**Conclusions/Significance:**

Our results suggest that CWP and its associated features share a genetic predisposition but that they are relatively distinct in their environmental determinants.

## Introduction

Chronic widespread musculoskeletal pain (CWP) is defined as “pain on the left side of the body, pain on the right side of the body, pain above the waist, pain below the waist and axial skeletal pain (cervical spine or anterior chest or thoracic spine or low back) for at least 3 months” [1]. It represents a key feature of the fibromyalgia (FM) syndrome which has a reported prevalence in the general population of approximately 15% [2,3]. In addition, high comorbidity of CWP with conditions such as chronic fatigue syndrome, headache, depression, and anxiety have been reported [4,5]. CWP not only causes profound individual suffering and disability in activities of daily living but is also associated with high health care utilization and increased health care costs [6]. Knowledge about the causes of CWP remains very limited but current research suggests that the pathology and its somatic expression appear to be influenced by genetic susceptibility and a variety of behavioral and psychosocial factors [7–9].

Quantitative and molecular genetic studies have provided consistent evidence for a genetic influence on CWP (heritabilities of up to 58%) and for a common genetic basis in multisite musculoskeletal pain [9,10–11]. Multivariable twin analyses investigating the importance of genetic factors on the etiology of CWP and its comorbidites (e.g., chronic fatigue, irritable bowel, anxiety and depression) have found evidence of unmeasured genetic and family environmental factors underlying the co-occurrence of all these conditions [12–13]. A recent study conducted by our research group (N = 3,266 female twins) explored the etiological structure of CWP and its psycho-affective correlates and found that the co-occurrences between CWP, anxiety, depression, emotional instability, and emotional intelligence could be explained by two distinct latent factors– a more sensory and a more affective one—which show a common, genetically determined underlying factor [11]. In summary, these findings point towards a heterogeneous etiological structure underlying CWP and its correlates, wherein physical, psychiatric and premorbid affective traits influence the development of the disorder.

Despite recent research efforts, our understanding of the commonly reported features of CWP such as fatigue, and/or symptoms of depression remains limited [14–15]. Fatigue in CWP patients has been described as chronic, abnormal and non-functional, and of complex or unknown etiology [16]. Reciprocal relationships between chronic fatigue, depression, physical incapacity and CWP have been suggested leading to a vicious cycle of poor mental and physical health [17]. Expression and levels of these CWP-associated symptoms seem to be influenced by hormonal alterations. The observations that prevalence of such symptoms is greater in women than men, and that the incidence of early menopause may be higher in FM patients compared to rheumatoid arthritis patients and healthy controls [18], has lead to the hypothesis that sex hormones may be involved in the pathogenesis of FM [19–20]. This is supported by the fact that women report an increase and worsening of generalized pain and fatigue when undergoing menopause [21,18]. Furthermore, some studies of women with FM show a correlation between low cortisol levels, pain and depression [22–23]. In this context, dehydroepiandrosterone sulfate (DHEAS)—an endogenous steroid hormone,—has been associated with the maintenance of FM symptomatology [21,22,24]. DHEAS directly influences testosterone production in the adrenal gland and the levels of androgens have been shown to decrease in pre- and postmenopausal women and to be inversely related to perceptions of stress and cortisol levels [21]. In a small cross sectional observational study of 17 women with FM and 19 healthy controls, Pegado and colleagues, for example, observed a tendency towards lower DHEAS levels in the FM group and a positive correlation between DHEAS and the pressure pain and tolerance [21]. Our recent work, using extensive and agnostic metabolomics screening in TwinsUK suggests that several DHEAS –related hormones, namely, androsterone sulfate, epiandrosterone sulfate, and DHEAS are strongly associated with CWP variation, in both the twin sample and independently replicated in a second group, the KORA study [25]. We found that the risk of CWP increased with a decrease in these hormones levels, and that with the latter appear to fall secondary to CWP rather than lying within the causal pathway to CWP. Multivariate genetic twin analyses were conducted to understand better the correlational structure and underlying etiological mechanisms in CWP and its common related biopsychological features. More specifically, the contributions of depression, fatigue, and serum DHEAS concentrations measured by automated electrochemiluminescence immunoassay in CWP disease expression were investigated by taking into account the inter-correlations among the phenotypes using a sample of unselected British female twin volunteers from the TwinsUK register.

## Material and Methods

### Participants

Participants were from the NIHR BRC BioResource TwinsUK, a nation-wide registry of female (83%) and male volunteer twins in the United Kingdom, with about 12,000 registered individuals [26,27]. The registry has an equal number of monozygotic (MZ) and dizygotic (DZ) twins which are predominantly middle-aged and older. The registry was started in 1993 and over the last 22 years extensive questionnaire, biomedical and clinical data have been collected. The primary focus of study has been the genetic basis of healthy aging processes and complex diseases, including cardiovascular, metabolic, musculoskeletal, and ophthalmologic disorders. For more detailed information regarding the cohort and the recruitment process see [26]. The cohort has been shown to be generalizable to UK population singletons for a wide variety of musculoskeletal, CVD, and metabolic traits, as well as lifestyle factors [28]. The present study received approval from the ethics committee at St. Thomas´ Hospital, and written consent was obtained before data collection. The research followed the tenets of the Declaration of Helsinki.

The study sample included all individuals who had CWP, and information on the other phenotypes of interest available. CWP data were available for a total of 3,266 female individuals. This study represents a sub-study of a larger project focusing on the epidemiology of CWP in females, hence no information regarding CWP was obtained for males at this point. Matching information on fatigue, depression and DHEAS were available for 463 twin pairs, comprising 219 MZ pairs, and 244 DZ pairs. Twin pairs where one twin had data but the co-twin did not have data (treated as missing values) were also included [29]. Individuals having conditions with known somatic causes of pain such as fracture, cancer, rheumatoid arthritis, and defined neuropathic causes of pain were excluded from the study. Zygosity of twins was assigned using a standard questionnaire and was confirmed with multiplex DNA genotyping and, more recently, genetic association markers on DNA obtained from venous blood samples.

### Measures

#### CWP

The London Fibromyalgia Epidemiology Symptom Screening Questionnaire (LFESSQ) was used to screen for self-reported CWP [30]. This 6-item questionnaire was originally designed to screen for FM in the general population and in specific patient groups and includes 4 items relating to widespread pain, and 2 items relating to fatigue. For the assessment of CWP, the four items pertaining to the “pain subscale” and asking about pain left and right of body and above and below diaphragm lasting at least 7 days in the previous 3 months were considered. In order to classify an individual as having CWP, participants had to respond “yes” to all four pain items with either both a right- and left-side positive response or a positive response for pain at both sides. The utility of this phenotype assessment is supported by the contribution these twins have made to previous studies, such as the genome-wide association meta-analysis conducted by Peters and colleagues [31] or the twin studies by Burri and colleagues [32,33].

#### CWP Related Biopsychological Features

Blood samples were collected from each of the study participants either after overnight fasting or non-fasting. Serum concentrations of DHEAS were measured by automated electrochemiluminescence immunoassay “ECLIA” (Roche Diagnostics, Mannheim, Germany). The intra- and interassay coefficients of variability were 2.3% and 4.4%, respectively.

Fatigue can be distinguished as peripheral muscle fatigue and central fatigue and previous reports have suggested that both subjective ratings and objective evaluation of fatigue measures (e.g., using surface electromyography) should be assessed [34]. However, this is relatively impractical for population samples and because subjective experiences might have a higher relevance for subjective well-being and impairment of quality of life, only subjective ratings of perceived fatigue were considered. For this, 2 items from the LFESSQ relating to fatigue were used to assess chronic fatigue. Screening positive for chronic, debilitating fatigue required a “yes” response to both fatigue items.

The diagnosis of depression was obtained using the depression module of the Composite International Diagnostic Interview (CIDI) questionnaire according to the DSM-IV criteria for major depression disorder (MDD) and was handled as a dichotomous variable in all analyses [32,35]. The CIDI interview included the three STEM questions (e.g. “Have you ever in your life had a period lasting several days or longer when most of the day you felt sad, empty or depression?”). Participants positively answering one of the three questions were given the depression module to fill in and were classified as having depression if five out of nine depressive symptoms persisting for two weeks or longer were present. Symptoms included dysphoric mood or anhedonia, weight gain/loss or appetite disturbance, insomnia or hypersomnia, psychomotor agitation or retardation, fatigue or loss of energy, feelings of worthlessness or excessive guilt, diminished ability to concentrate or think clearly, and recurrent thoughts of death or suicide [35].

### Twin Analyses

The classical twin design was used to conduct univariate and multivariate analyses of genetic and environmental influences on the variance and covariance of CWP, fatigue, depression and DHEAS. The twin method is based on the assumption that twins reared together resemble each other due to the additive effects of shared genes or shared environmental factors. MZ twins further share around 100% of all segregating genes whereas DZ twins share—on average—50% of their segregating alleles. For both univariate and multivariate analyses, structural equation modeling with full information maximum-likelihood estimation was used and phenotypic variance and covariance was decomposed into additive genetic (A), dominant genetic (D), shared environmental (C), and non-shared environmental (E) etiologies [36–39]. All observed variables were adjusted for age in the models, and standardized path coefficients with the 95% confidence intervals (CIs) were obtained. To handle missing values, full information maximum likelihood was used [29]. Under a missing at random assumption, full information maximum likelihood was able to give preferred parameter estimates [29]. Data handling and all statistical analyses were conducted using R statistical software, version 3.1.2 (R Core Team, 2014). Genetic analyses were conducted using the R package “OpenMx”.

For the calculation of univariate heritabilities of each phenotype, structural equation modeling (SEM) was used. Univariate analyses were extended to multivariate twin analyses to investigate the relative importance of genetic and environmental factors in the phenotypic associations between our four phenotypes of interest—DHEAS, fatigue, depression, and CWP. The multivariate genetic model estimates genetic and environmental mediation of the phenotypic correlation between the phenotypes of interest. Central to the analysis is the genetic correlation, which is the extent to which genetic effects on one variable are correlated with genetic effects on another variable, indicating pleiotropy. First, MZ and DZ cross-trait cross-twin correlations (CTCT) were examined. A CTCT correlation is a correlation between trait A in a twin and trait B in the co-twin. The phenotypic associations between the observed variables were partly decomposed into A and D if the phenotypic associations between the observed variables were higher in MZ compared to DZ twin pairs. In cases where the phenotypic associations in MZ twin pairs were similar or equal to those in DZ twins, the phenotypic associations were decomposed into C. In cases where no phenotypic associations between the observed variables within a twin pair could be detected, the phenotypic associations were decomposed into E.

Next to indicate the best fitting, and theoretically most acceptable and parsimonious model, the following four-step analyses were conducted. First, the full ACE and ADE Cholesky models were compared with the fully saturated model since C and D cannot be estimated in the same model [37,38]. Model comparison also allows testing of the assumption that mean and variance of each observed phenotype is equal across twin order and zygosity—a prerequisite for multivariate twin modeling [38]. In the fully saturated model, the variance-covariance matrix was treated as a free parameter equal to the sample variance-covariance matrix. In the full Cholesky model, mean and variance of each observed phenotype were modeled to be equal across twin order and zygosity [37]. Thus, the assumption was met if the log likelihood ratio test showed no significant differences between the full Cholesky model and the fully saturated model [37]. Second, the full ACE or ADE Cholesky model was compared to the AE Cholesky model to test whether elimination of C or D factors was possible.

The Cholesky decomposition provides the correlations between the four independent genetic and environmental factors and decomposes the variance of the four phenotypes into distinct additive genetic and distinct non-shared environmental effects, providing the fullest potential explanation of the data. Here, the expected variance-covariance matrix is parameterized in terms of n (= number of variables) latent factors. The independent pathway model is a submodel of the Cholesky model. It generally tests whether the covariance between the four phenotypes can be explained by a single shared genetic and shared environmental factor. An independent pathway model would suggest that all phenotypes share common etiological factor [37]. A common pathway model suggests that a latent phenotype underlies the four phenotypes, which in turn is influenced by shared genetic and shared unique environmental factors [37].

Third, the AE Cholesky model was compared to an AE independent pathway model and an AE common pathway model. Fourth, on the basis of the most plausible AE model among the AE Cholesky, AE independent pathway, and AE common pathway models, we made sub-models eliminating parameters and latent variables and compared these sub-models to the full ACE or ADE Cholesky model. Model comparisons were carried out on the basis of the likelihood ratio test and Akaike information criterion (AIC), with a smaller AIC indicating a better balance of model fit to the observed data and parsimony [38].

## Results

The sample selected from TwinsUK was entirely female. The mean age was 58.4 years (*SD* 11.1, range 26 to 82 years; Table 1). In this sample the prevalence of CWP was 19.6% and of depression was 30.1%. No significant differences in the phenotypes of interest could be detected between MZ and DZ twins (Table 1).

**Table 1:** Characteristics of the study group; overall and by zygosity.

	Overall	MZ	DZ	P-value
Age	58.39 (11.08) 26–82	58.63 (11.50) 26–82	58.14 (10.67) 27–80	0.602
DHEAS (umol/L)	3.10 (1.93) 0.06–12.23	3.16 (2.00) 0.13–12.23	3.03 (1.87) 0.06–10.95	0.683
Fatigue	1.55 (1.29) 0–4	1.56 (1.27) 0–4	1.55 (1.31) 0–4	0.234
CWP	126 (19.62)	62 (19.5)	64 (19.75)	0.535
Depression	193 (30.06)	97 (30.5)	96 (29.62)	0.552

Age, DHEAS, fatigue are given as mean (SD), range; CWP and Depression as N = sample size (%)

Overall sample = 642 individuals, 463 full pairs. MZ = 318 individuals, 219 full pairs; DZ = 324 individuals, 244 full pairs.

No significant differences between the MZ and DZ could be detected.

MZ = monozygotic twins; DZ = dizygotic twins; CWP = chronic widespread pain; DHEAS = dehydroepiandrosterone sulfate.

### Phenotypic Correlation and Phenotypic Heritability

First, the phenotypic correlations and CTCT correlations between the four phenotypes were examined. The highest phenotypic correlation was seen between fatigue and CWP (r = 0.45) (Table 2). DHEAS showed a significant negative correlation with CWP (-0.23) but was not correlated with either depression or fatigue. There were statistically significant correlations between all the other phenotypes. High to moderate heritabilities for all phenotypes were obtained, with DHEAS showing the highest estimate (74.2%; 95% CI 65.8% to 80.5%), followed by CWP (70.8%; 95% CI 44.1% to 87.8%), depression (49.9%; 95% CI 19.0% to 74.1%) and fatigue (42.6%; 95% CI 24.7% to 57.0%). Next, the CTCT correlations were calculated to estimate the extent to which the phenotypic correlations between the four phenotypes are mediated by genetic and environmental influences. Analyses revealed that CTCT correlations were generally higher in MZ compared to DZ twins, indicating that the phenotypic covariance among the three symptoms is largely due to shared genetic factors. In other words, higher CTCT correlations in MZ twins suggest that genetic factors underlie the correlation between the four phenotypes (Table 3).

**Table 2:** Phenotypic associations between variables of interest (N = 642 individuals).

	DHEAS	Depression	Fatigue	CWP
DHEAS	1			
Depression	-0.020	1		
Fatigue	0.055	0.286**	1	
CWP	-0.226**	0.321**	0.454**	1

CWP = chronic widespread pain; DHEAS = dehydroepiandrosterone sulfate.

Pearson correlations between continuous variables (DHEAS and fatigue); tetrachoric correlations between dichotomous variables (depression and CWP); biserial correlations between continuous and dichotomous variables.

* p<0.05;

** p<0.01

**Table 3:** Cross twin cross trait correlations (CTCT with 95% confidence interval) between the traits of interest.

CTCT (95%CI)				
	DHEAS	Fatigue	Depression	CWP
DHEAS	0.805 (0.794 to 0.850)			
	0.501 (0.373 to 0.611)			
Fatigue	0.080 (-0.071 to 0.228)	0.513 (0.388 to 0.619)		
	-0.033 (-0.204 to 0.141)	0.094 (-0.088 to 0.270)		
Depression	-0.055 (-0.198 to 0.091)	0.234 (0.083 to 0.375)	0.597 (0.495 to 0.686)	
	-0.082 (-0.242 to 0.081)	-0.090 (-0.264 to 0.090)	-0.067 (-0.231 to 0.101)	
CWP	-0.235 (-0.363 to -0.098)	0.316 (0.175 to 0.445)	0.353 (0.220 to 0.473)	0.717 (0.642 to 0.779)
	-0.195 (-0.341 to -0.040)	0.224 (0.054 to 0.382)	0.134 (-0.028 to 0.288)	0.376(0.235 to 0.502)

MZ CTCTs are displayed in the upper row, DZ CTCTs in the lower row.

MZ = 219 pairs; DZ = 244 pairs

CWP = chronic widespread pain; DHEAS = dehydroepiandrosterone sulfate; MZ = monozygotic; DZ = dizygotic.

### Multivariate Genetic Analyses

**Fig 1:**
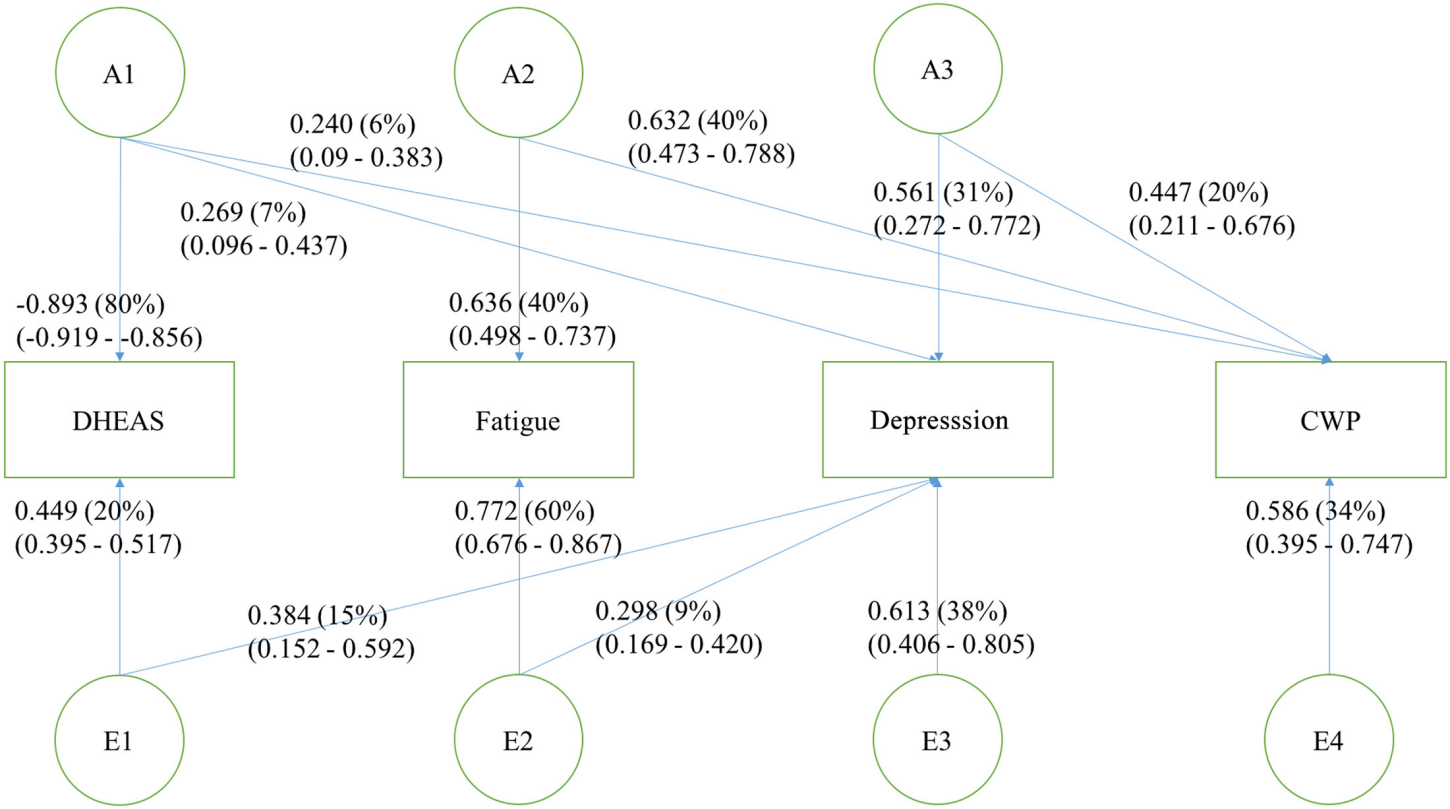
Path diagram of the best fitting AE Cholesky model depicting the sources of covariance between CWP, DHEAS, depression, and fatigue. The variance of 219 monozygotic (MZ) and 244 dizygotic (DZ) twin pairs was decomposed into two additive genetic (A1–A2) and an unshared environmental (E1) factor. Standardized factor loadings with 95% confidence interval (CI) and variances for the phenotypes are displayed.

Multivariate genetic analyses were conducted to investigate the genetic and environmental structure among DHEAS, fatigue, depression, and CWP. Model comparison among the fully saturated model, full ACE Cholesky model and full ADE Cholesky model were performed (Table 4). Of the full models, the ADE Cholesky model provided the best balance of fit and parsimony of the observed data, based on the AIC. Subsequently, model comparison among the full ADE Cholesky model, the AE Cholesky model, the AE independent pathway models, and the AE common pathway models were performed (Table 5). On the basis of the log likelihood ratio test, an AE Cholesky model provided the best fitting and most parsimonious model (Table 5; Fig 1). According to that model, the variance and covariance in the phenotypes was decomposed into three distinct additive genetic factors and four distinct non-shared environmental factors. We calculated percentage of genetic variance in phenotypic variance of each variable (100 x genetic variance/ phenotypic variance). Two additive genetic factors loading heavily on CWP could be detected (A2 explaining 40% of the phenotypic variance and A3 20%), with A2 also explaining 40% of phenotypic variance in fatigue and A3 explaining 31% of phenotypic variance in depression. The factor loading most heavily on DHEAS showed only small loads on the other phenotypes and no load at all on fatigue (Fig 1). Similarly, we calculated the percentage of non-shared environmental variance in phenotypic variance of each variable (100 x non-shared environmental variance/ phenotypic variance). The non-shared environmental factor explaining 20% of phenotypic variance in DHEAS did not show any effect on fatigue and CWP. Interestingly, only one distinct non-shared environmental factor loading on CWP could be detected, explaining 34% of phenotypic variance in CWP, hence suggesting that the association between CWP and the other phenotypes is due to only genetic factors (Fig 1).

**Table 4:** Results of the model comparison between the fully saturated, the full ACE Cholesky, and the full ADE Cholesky modeling.

	Difference of log likelihood	Difference of degree of df	P-value	AIC
The fully saturated model	Base	Base	Base	-394.675
The full ACE Cholesky model	59.013	58	0.438	-451.661
The full ADE Cholesky model	57.562	58	0.491	-453.112

MZ (N = 219 pairs) and DZ (N = 244 pairs).

A = additive genetic factors; C = shared environmental factors; D = non-additive genetic factors; E = non-shared environmental factors; AIC = Akaike information criterion; df = degree of freedom; MZ = monozygotic; DZ = dizygotic.

The most suitable model by AIC is the ADE Cholesky model (in bold)

**Table 5:** Results of the model comparison among the full ADE Cholesky model and sub-models.

	Difference of log likelihood	Difference of df	P-value	AIC
Full ADE Cholesky model	Base	Base	Base	-453.112
AE Cholesky model	3.959562	10	0.949	-469.153
AE independent pathway model with one genetic or environmental latent variables common to observed variables	19.69748	14	0.139	-461.415
AE common pathway model with one phenotypic latent variables common to observed variables	25.41869	17	0.085	-461.694
AE independent pathway model with two genetic or environmental latent variables common to observed variables	13.45777	6	0.036	-451.655
AE common pathway model with two phenotypic latent variables common to observed variables	15.53236	10	0.113	-457.58
**Cholesky AE model** (**see** Fig 1)	**10.99799**	**17**	**0.856**	**-476.114**

Sample: MZ (N = 219 pairs) and DZ (N = 244 pairs).

A = additive genetic factors; C = shared environmental factors; D = non-additive genetic factors; E = non-shared environmental factors; AIC = Akaike information criterion; df = degree of freedom; MZ = monozygotic; DZ = dizygotic.

The most suitable model is shown in bold, having the lowest AIC

Overall fatigue, depression and DHEAS accounted for 65.7% of the variance in CWP with the Cholesky model revealing that the genetic variance of CWP was explained by the covariation of the three phenotypes, with 9% shared with DHEAS, 61% with fatigue, and 30% with depression. The highest non-shared environmental correlations (i.e., correlations measure shared by the variables´ random environmental effects, with respect to the individual) could be observed between depression and DHEAS (r_E_ = 0.49), followed by depression and fatigue (r_E_ = 0.38; Table 6). None of the other phenotypes correlated environmentally with each other. Furthermore, high genetic correlation between CWP and fatigue (r_G_ = 0.78) and depression (r_G_ = 0.62) could be detected, as well as more moderate genetic correlations between CWP and DHEAS (r_G_ = -0.29) and between DHEAS and depression (-0.43; Table 6).

**Table 6:** Environmental and genetic correlations between the phenotypes of interest.

CTCT (95%CI)				
	DHEAS	Fatigue	Depression	CWP
DHEAS	1			
	1			
Fatigue	0	1		
	0	1		
Depression	-0.433	0	1	
	0.491	0.381	1	
CWP	-0.296	0.780	0.625	1
	0	0	0	1

Estimates were derived from the best fitting AE Cholesky model in MZ (N = 219 pairs) and DZ (N = 244 pairs).

Genetic correlations are displayed in the upper row, environmental correlations in the lower row

Abbreviations: CWP = chronic widespread pain; DHEAS = dehydroepiandrosterone sulfate; MZ = monozygotic; DZ = dizygotic.

## Discussion

Although the co-occurrence of CWP with symptoms of fatigue and depression has been reported frequently, the etiology of each symptom and how they relate to one another remains poorly understood. In the present study we used the classical twin design to investigate the link between CWP and its biophysiological correlates and found a strong association between fatigue and CWP, with genetic factors of chronic fatigue explaining 60.8% of genetic variance of CWP. Additionally we report evidence for a strong genetic link between CWP and fatigue, as well as depression. The association between DHEAS and fatigue reported previously in small clinical studies was not confirmed in our study. However, moderately strong association between DHEAS and CWP and DHEAS and depression were observed. In addition, one distinct non-shared environmental factor loading specifically on CWP—but not on any of the other phenotypes—was found, suggesting that the association between CWP and the other phenotypes is mediated by genetic factors alone. These findings provide further insights into the etiologic nature of CWP and its clinical features. One might speculate that the environmental factor might represent adverse early life events such as maternal separation but we are not in a position to test this hypothesis at present.

### Heritability of CWP and its Clinical Features

The best fitting models from the univariate analyses showed that all phenotypes were heritable (h^2^ ranging from 42.6% to 74.2%), and comprised only A and E factors (including measurement error) to explain phenotypic variance. The highest heritability was found for DHEAS with an estimate of 74.2%. Biological and anatomical phenotypes generally show higher genetic influences as compared to psychological or behavioral traits. Previous reports, such as the study by Yildiz or Nestler and colleagues reported lower heritabilities of DHEAS of 43% and 60% respectively [40,41]. Similarly, the heritability of CWP in the present study (70.83%) was higher than previously reported estimates (e.g. 58% and 54% by Kato *et al*. and Markkula *et al*., respectively) [9,42]. These differences might be the result of restricted sample size and/or un-adjustment for potential additional covariates. For example, in the previous study from TwinsUK a significant correlation of CWP with fat mass was seen using a slightly different sample [25]. In terms of chronic fatigue, the estimated heritability of 42.6% matched previous reports (ranging from 30% to 51%). Overall, and in line with previous reports, we found no evidence of environmental influences shared by siblings on individual differences in any of the phenotypes. Instead, the type of environmental influence that was important was entirely of a non-shared nature and, in case of CWP and DHEAS, considerably smaller than the genetic effects.

### Common Genetic and Specific Environmental Influences on the Covariation between CWP, Fatigue and Depression

Although exploration of the correlates of CWP has been the target of substantial epidemiologic research in the past, there have been very few multivariate studies especially using genetically sensitive designs. The genetic correlation results from our genetic analyses describe the extent to which genetic effects on one variable are correlated with genetic effects on another. Genetic correlation between CWP and fatigue was 0.78 and between CWP and depression was 0.63. There was no evidence of a common non-shared environmental etiology to CWP or any of the other phenotypes. Overall these findings suggest that two of the major features of CWP—chronic fatigue and symptoms of depression—are related to CWP largely for genetic reasons, thus we tentatively speculate that they may not be *consequences* of the chronic pain condition *per se* but rather symptomatologically related. In other words, the chronic fatigue often observed in CWP patients may in fact not be due to the general effect that chronic pain can have on energy levels or due to side-effects of medication but seem to be part of the clinical syndrome. This is further supported by the fact that fatigue accounted for a larger proportion of the genetic variance in CWP (61%) than any of the other phenotypes (30% explained by depression and only 9% by DHEAS). Again, we reiterate that replication of the present findings in an independent sample will add further weight to the findings.

Our genetic modeling supports previous findings which illustrate that features of CWP are relatively etiologically distinct from each other—particularly in terms of environmental factors. This is not only supported by the heterogeneity in phenotype specific non-shared environmental factors that we detected but also by the finding that both the common and independent pathway models (both assuming a common underlying structure to the phenotypes, either a latent phenotype or a unanimously shared overarching etiologic structure) provided poorer fits to the data than the Cholesky model, indicating that the different CWP features are not explained by a single underlying disease entity.

### CWP-Related Chronic Fatigue and DHEAS Dysregulation

Dysregulation of DHEAS has been proposed to play an etiologic role in the maintenance of FM symptomatology by modulating inflammatory responses [43,44]. The endogenous steroid hormone has further been ascribed a pivotal role in fatigue, general well-being, and depression [45,46]. Study results, however, are fairly inconsistent. Numerous placebo controlled, randomized clinical trials have investigated the use of DHEA substitution in a variety of clinical populations using fatigue as the main outcome measure. While some reported a positive effect on psychological well-being, mood, and fatigue [47–49], others were unable to detect such an influence [45,50–51]. Interestingly, a strong placebo effect could be observed in many of the unsuccessful clinical DHEAS trials, suggesting a potential role of psychotherapy to be able to provide considerable benefits in treating chronic fatigue in these patients. We did not observe a link between DHEAS and fatigue in our study, thus mirroring the discrepant study reports that have failed to produce consistent proof of association of DHEAS in chronic fatigue (e.g., [48–49,52]). Here, it has to be noted that the majority of the past studies were small while here we report findings in one of the largest samples so far. Many studies have investigated the etiology and pathogenesis of chronic fatigue, and different biological mechanisms have been proposed in the past, with DHEAS and the HPA axis being one candidate. Nowadays, the etiology and pathogenesis is generally believed to be multifactorial [47], with symptom explanations being sought in viral infections, immune dysfunction, neuroendocrine responses, genetic factors, personality, (neuro)psychological processes, etc.—to name a few [53]. It has been further proposed that the development of the symptoms is due to distinctive categories of predisposing, precipitating, and perpetuating factors and that the presence of one or more factors is conditional but insufficient [54]. Given the absence of a link between DHEAS and fatigue in our study—one of the largest to date—it is likely that the mechanisms promoting symptoms of fatigue in CWP and FM are unrelated to the HPA axis and DHEAS dysregulation and that other sources– possibly psychological—have to be considered.

We observed moderate negative genetic associations between DHEAS and CWP and DHEAS and depression. Here, the association of DHEAS and CWP was exclusively due to a shared genetic basis, again suggesting—similar to CWP and fatigue—that the lower DHEAS levels in individuals suffering from chronic pain are not a consequence of the condition but may be even viewed and used as a potential CWP endophenotype or biomarkers in future studies [54,55].

### Environmental Etiology

While the overall study goal was to describe the genetic landscape of CWP using a set of biopsychiological features, our results also inform on the environmental influences which sensitive designs such as the twin model can help disentangle. Most notable is our finding of a specific non-shared environmental factor explaining 34.3% of the variance in CWP that shows no influence on any of the other CWP covariates. If the sources of the variance in CWP are indeed environmental factors, the source of this influence would appear to be an especially good target for intervention but more fine-grained studies will be needed to identify precise environmental predictors.

### Limitations

Some limitations should be noted. Our findings require independent replication to ensure that they are generalisable to other populations. However, compared to the vast number of previously conducted studies—especially of a clinical nature—the sample size is good. Second, the screening tool used in this study did not include a clinical evaluation of CWP; instead, presence of CWP was determined using short, validated self-report questionnaire. This approach may be variable with regard to adherence to a strict set of diagnostic criteria for CWP, thus our results should be interpreted with this caution in mind. However, questionnaire-based screening has been suggested as useful in general population surveys, with high positive predictive value and test-retest reliability [30]. Similarly, self-report was used to assess depression, rather than relying on a clinical diagnoses. But because the questions assessing the various symptoms were independent from each other, and the subjects were unaware of the hypothesis being tested, the correlation among the phenotypes found in our study reflects real trait co-occurrence rather than an artifactual one resulting from overlapping definition criteria for depression, fatigue conditions, and CWP. Third, our analyses included female twins only and, therefore, results cannot be generalized to adult males. Sex differences in genetic and environmental variances in CWP, depression and fatigue have previously been reported [56,57]. Fourth, the association between the symptoms may vary with age and although we controlled for the main effects of age in structural equation modeling, extrapolation of the results to other age groups is not possible and a longitudinal design will need to be employed to investigate age-dependent phenotypic variation. Similarly, the higher prevalence of CWP and depression found in our study compared to previous ones might be the result of the preponderance of females of middle age in the TwinsUK registry. Finally, only one DHEAS sample per individual for one time point was available. Two inherent difficulties with the study of hormones is their variability over time and the determination of whether an effect is primary or secondary [58]. Hence, longitudinal studies should be conducted in the future.

## Conclusions

In conclusion, our results suggest that the associations between CWP and fatigue and depression are due to shared genetic factors alone, indicating that neither the shared nor unshared environments contribute to their co-occurrence. Similarly, a genetic relationship between DHEAS and CWP and depression were found. We do not find an association between DHEAS and fatigue. Overall, our findings support previous studies in that features of CWP such as depression and fatigue are etiologically distinct from each other on an environmental level but that they share a genetic base which accounts for their clinical co-occurrence. Our findings highlight an avenue for future research that may provide additional utility in characterization of the complex etiology and clinical presentation of CWP.
